# Acid–base balance and respiratory mechanics during robotic-assisted surgery: an observational study

**DOI:** 10.1007/s11701-026-03392-8

**Published:** 2026-04-21

**Authors:** Matteo Pitimada, Davide Chiumello, Alessandro Monte, Isabella Fratti, Federica Festa, Tommaso Pozzi, Silvia Coppola

**Affiliations:** 1 Department of Anesthesia and Intensive Care, ASST Santi Paolo e Carlo, San Paolo University Hospital Milan, Milan, Italy; 2 Department of Health Sciences, University of Milan, Milan, Italy; 3 Coordinated Research Center on Respiratory Failure, University of Milan, Milan, Italy

**Keywords:** Robotic-assisted surgery, Respiratory mechanics, Mechanical power, Acid-base equilibrium, Pneumoperitoneum

## Abstract

To evaluate changes in acid-base equilibrium in plasma and urine according to the Stewart’s approach, in gas exchange and in respiratory mechanics during robotic-assisted surgery. Prospective observational study on patients undergoing robotic-assisted surgery. Acid-base equilibrium in plasma and urine was measured after 10 min from general anesthesia induction (T0), after 2 h from pneumoperitoneum administration (T1) and after 10 min from pneumoperitoneum interruption (T2). In the same timepoints respiratory mechanics, gas exchange and hemodynamics variables were measured. Seventy-three patients were enrolled. The apparent strong ion difference did not change during the whole study. Urine sodium and chloride concentration did not change throughout the study, although sodium absolute excretion decreased. Urinary anion gap increased from 35 [26–42] at T0 to 39 [28–53] mEq L^− 1^ at T2. At T1, a significant increase in arterial carbon dioxide partial pressure (45 [42–51] vs. 41 [37–45] mmHg) as well as a consequent reduction in arterial pH (7.36 [7.3–7.38] vs. 7.41 [7.37–7.44]) compared to T0 was found. At T2, arterial carbon dioxide partial pressure and arterial pH decreased but never returned to baseline. The behavior of acid-base equilibrium in plasma and urine was independent from fractional sodium excretion. Pneumoperitoneum did not alter plasma strong ion difference or urinary electrolytes concentration, although it induced respiratory acidosis with acidemia. The absence of any compensatory change in urinary electrolytes was probably related to the hemodynamic effect of pneumoperitoneum on the kidneys, resulting in a reduction in absolute excretion of sodium.

**Supplementary Information:**

The online version contains supplementary material available at 10.1007/s11701-026-03392-8.

## Introduction

Robotic-assisted surgery is an increasingly used laparoscopic technique, particularly for colorectal, gynaecological, thoracic and urological procedures, due to faster recovery, improved outcomes and reduced pain [1].

Robotic-assisted procedures require carbon dioxide insufflation to establish pneumoperitoneum. The resulting increase in intra-abdominal pressure leads to cranial displacement of the diaphragm, reduction of functional lung volumes, and development of dependent atelectasis, with consequent impairment of respiratory compliance and gas exchange [1, 2].

Prolonged ventilation in the setting of altered respiratory mechanics may exacerbate ventilator-induced lung injury [3]; therefore, PEEP and moderate-to-low tidal volumes are commonly applied to limit lung damage [4]. More recently, mechanical power has been proposed to quantify the interaction between pneumoperitoneum-induced mechanical changes and ventilator settings, similarly to the ARDS framework [5, 6].

Pneumoperitoneum can also decrease cardiac output by raising intra-abdominal pressure, thereby reducing venous return and increasing afterload, often with minimal changes in arterial pressure [7, 8]. Renal perfusion may decline markedly (up to ~ 40%), leading to reduced urine output and creatinine clearance. Increased intra-abdominal pressure can impair renal venous outflow, lowering renal perfusion pressure; this may be more injurious than reduced arterial inflow because renal capsule constraints and impaired lymphatic drainage limit tissue decompression [9]. These global and regional hemodynamic alterations can trigger a compensatory neuroendocrine response involving the release of dopamine, adrenaline, noradrenaline, renin and cortisol. This *stress response* is frequently encountered during all types of surgery and could be exacerbated during pneumoperitoneum application due to the reduction in venous return and in renal venous outflow following the increase in intra-abdominal pressure. This leads to increased sodium retention by the kidneys [10].

Carbon dioxide (CO₂) used for pneumoperitoneum can be absorbed through the peritoneum, causing hypercapnia and respiratory acidosis [11]. The magnitude of acidosis depends on factors such as insufflation duration, intra-abdominal pressure, number of ports, age and pre-existing pulmonary disease [12]. From a physiological standpoint, sustained hypercapnia is expected to activate renal acid excretion mechanisms, primarily through increased ammoniagenesis, which requires parallel chloride handling to preserve electroneutrality [13]. As so, the resulting effect of pneumoperitoneum on kidney physiology would be to induce sodium retention due to the increase in intra-abdominal pressure and to increase chloride excretion to compensate for hypercapnia.

Acid–base disturbances in the perioperative setting are traditionally interpreted using the Henderson–Hasselbalch framework; however, this model does not fully account for variations driven by changes in electrolytes or weak non-volatile acids. The physicochemical approach proposed by Stewart integrates these variables and may therefore provide a more comprehensive interpretation of perioperative acid–base alterations [14]. To date, no clinical or experimental studies have specifically assessed the effects of robotic-assisted surgery on acid-base equilibrium.

The primary aim of this study was to evaluate changes in the acid-base equilibrium in plasma and in urine according to the Stewart’s approach, along with changes in respiratory mechanics and gas exchange induced by pneumoperitoneum application during robotic-assisted colorectal and gynecological surgery. Our secondary aim was to investigate the differential effect of an increased intra-abdominal pressure, which may increase sodium retention, and of an increased arterial CO_2_, which may increase chloride excretion, stratifying our study population according to the sodium retention behaviour in terms of higher and lower sodium fractional excretion.

## Materials and methods

### Study population

This study was conducted from December 2023 to December 2024 in the operating surgical department of the ASST Santi Paolo Carlo, San Paolo University Hospital in Milan, Italy. All consecutive patients with ASA I-III who were undergoing elective robotic-assisted colorectal or gynecological surgery were considered eligible for the study. Patients were excluded if they had chronic obstructive pulmonary disease, documented pulmonary bullae, a history of lung surgery, glaucoma or a body mass index higher than 35 kg/m^2^.

The study was approved by the ASST Santi Paolo e Carlo institutional review board (protocol number 104/2816 November 2023); informed consent was obtained in accordance with Italian regulations.

### Study protocol

A flow chart of the study protocol is presented in Figure S1. Before surgery began, patients were given intravenous fluids to compensate for preoperative fasting (1 mL/kg for each hour of fasting). Anesthesia was then induced using propofol (2 mg/kg), fentanyl (1.5–2 µg/kg) and rocuronium (1.2 mg/kg). After tracheal intubation, balanced anesthesia was maintained by administering a sevoflurane concentration to obtain bispectral index values between 40 and 50, alongside a remifentanil infusion between 0.02 and 0.20 µg/kg/min according to hemodynamic parameters. The degree of neuromuscular blockade was measured using the train-of-four (TOF) monitoring technique. Rocuronium was administered to maintain a TOF count of less than 1. Patients were monitored using routine physiologic monitoring, including a 3-lead electrocardiogram and pulse oximetry. Invasive arterial blood pressure monitoring and urinary catheter were set up immediately after anesthesia induction.

Mechanical ventilation was delivered using a Flow-i ventilator in volume-controlled mode, with standardized ventilatory settings applied to all patients as part of a protective intraoperative strategy: a tidal volume (V_T_) of 8 mL/kg of predicted body weight, an inspiratory to expiratory ratio (I: E) of 1:2 and a PEEP of 5 cmH_2_O, to standardize the effect of increased intra-thoracic pressure on renal hemodynamics. The inspired oxygen fraction (FiO_2_) was titrated to maintain a peripheral oxygen saturation greater than 92%. The respiratory rate (RR) was adjusted to obtain an end-tidal carbon dioxide concentration (EtCO_2_) between 32 and 38 mmHg. During surgery, the respiratory rate was adjusted in order to maintain an EtCO_2_ lower than 50 mmHg, which would correspond to an arterial carbon dioxide partial pressure (PaCO_2_) of 55–62, assuming a EtCO_2_/PaCO_2_ ratio of 0.8–0.9.

During surgery, patients received an intravenous infusion of Ringer Lactate solution (5 mL/kg/h) to compensate for insensible losses. A mean arterial pressure greater than 65 mmHg was maintained by fluid resuscitation (a bolus of Ringer‘s Lactate solution of 500 mL) in the presence of a pulse pressure variation greater than 12%. Otherwise, an infusion of norepinephrine was started.

Pneumoperitoneum was produced by insufflating CO_2_ into the abdomen to achieve an intra-abdominal pressure of 10 mmHg throughout the surgical procedure. Patients were positioned supine on in mild Trendelenburg (≤ 15° head-down tilt); no patient was exposed to extreme Trendelenburg positioning during surgery.

### Data collection

At enrollment anthropometric and demographic variables, as well as the American Society of Anesthesiologists (ASA) physical status classification and anamnestic data (previous history of hypertension, diabetes, coronary artery disease or cerebrovascular disease) were retrieved.

Ten minutes after general anesthesia was induced (T0), the following data were obtained:

- ventilatory setting: V_T_, inspiratory flow, RR, minute ventilation (V_E_), PEEP and FiO_2_;
- respiratory mechanics: peak pressure; plateau airway pressure and total PEEP were obtained by an end-inspiratory and end-expiratory hold maneuvers, respectively;
- hemodynamics: systolic, diastolic and mean arterial pressure, heart rate, vasopressor requirement;
- arterial blood gas variables: pH, arterial carbon dioxide partial pressure (PaCO_2_), arterial oxygen partial pressure (PaO_2_), actual bicarbonate concentration ([HCO_3_^−^]), base excess (BE); sodium (p[Na^+^]), potassium (p[K^+^]), calcium (p[Ca^++^]), chloride (p[Cl^−^]) and lactate (p[Lac^−^]) concentrations;
- plasma magnesium (p[Mg^++^]), phosphate, albumin and creatinine concentrations;
- urinary variables: sodium (u[Na^+^]), potassium (u[K^+^]) and chloride (u[Cl^+^]) concentrations; total amount of infused fluid and total diuresis. Differential diuresis was calculated as the amount of urine produced from the time the urine catheter was positioned to T0, from T0 to T1 and from T1 to T2.

Arterial blood gas analysis was performed using the RAPIDPoint 500 analyzer (Siemens Healthcare, Erlangen, Germany).

The same set of measurements was obtained 2 h after the start of pneumoperitoneum (T1) and 10 min after its interruption at the end of the surgery (T2).

### Derived variables

Plasma apparent strong ion difference (SID) was calculated as the difference between the sum of strong cations and the sum of strong anions in plasma [15]:

$$\begin{aligned} & Apprent~SID=\left( {p\left[ {N{a^+}} \right]+p\left[ {{K^+}} \right]+2 \times p\left[ {C{a^{++}}} \right]+2 \times p\left[ {M{g^{++}}} \right]} \right) \\ & - \left( {p\left[ {C{l^ - }} \right]+p\left[ {La{c^ - }} \right]} \right) \end{aligned}$$

The concentration of total weak non-carbonic acids (A_TOT_) was calculated as:

$${A_{TOT}}=0.28 \times \left[ {Albumin} \right]+1.8 \times \left[ {Phosphate} \right]$$

where albumin concentration is in g L^− 1^ and phosphate concentration is in mMol L^− 1^. Plasma effective SID was calculated as the sum of the charges provided by bicarbonate, albumin and phosphate, measured by an arterial sample [15]:

$$\begin{aligned} & Effective~SID=\left[ {Albumin} \right] \times \left( {0.123 \times pH - 0.631} \right) \\ & \quad +\left[ {Phosphate} \right] \times \left( {0.309 \times pH - 0.469} \right) \end{aligned}$$

where albumin concentration is in g L^− 1^ and phosphate concentration is in mMol L^− 1^. The strong ion gap (SIG), reflecting the amount of unmeasured anions, was calculated as the difference between apparent and effective SID [15].

Fractional excretion of sodium (FeNa) was calculated as:

$$FeNa=\frac{{pCr \times p\left[ {N{a^+}} \right]}}{{uCr \times u\left[ {N{a^+}} \right]}} \times 100$$

where pCr and uCr are plasma and urine creatinine concentration in mg/dL and p[Na^+^] and u[Na^+^] are plasma and urine sodium concentration [16].

Potassium and chloride fractional excretion were calculated similarly.

Absolute urinary electrolyte excretion was calculated by multiplying urinary electrolyte concentrations by urine output.

Urinary anion gap (UAG) was computed as:

$$UAG=u\left[ {N{a^+}} \right]+u\left[ {{K^+}} \right] - u\left[ {C{l^ - }} \right]$$

where u[K^+^] and u[Cl^−^] are urine potassium and chloride concentration [16].

Driving pressure was calculated as:

$$Driving~Pressure=Plateau~Pressure - total~PEEP$$

Respiratory system elastance was calculated as:

$$Respiratory~system~elastance=\frac{{Driving~Pressure}}{{{V_T}}}$$

Airway resistance was calculated as:

$$Airway~resistance=\frac{{Peak~pressure - Plateau~pressure}}{{\dot {V}}}$$

where V̇ is peak inspiratory airway flow. Airway resistance was calculated at the administered inspiratory flow.

Mechanical power (MP) was calculated as [5]:

$$\begin{aligned} & MP=0.098 \times {V_T} \times RR \\ & \times \left( {Peak~pressure - {\raise0.7ex\hbox{${Driving~Pressure}$} \!\mathord{\left/ {\vphantom {{Driving~Pressure} 2}}\right.\kern-0pt}\!\lower0.7ex\hbox{$2$}}} \right)\end{aligned}$$

Ventilatory ratio (VR) was calculated as [17]:

$$VR=\frac{{{V_E} \times PaC{O_2}}}{{0.1 \times IBW \times 40}}$$

where IBW is ideal body weight.

### Statistical analysis

The application of pneumoperitoneum and the stress response are known to reduce sodium excretion during surgery. Conversely, respiratory acidosis with hypercapnia is thought to increase urine ammonium and chloride excretion to maintain acid-base balance. In the absence of data in terms of urinary ammonium and chloride excretion during surgery with pneumoperitoneum, we based our sample size on the reported changes in urine sodium concentration changes. For the sample size calculation, the null hypothesis was that the mean change in urine sodium concentration between baseline and end of surgery would be zero. Assuming a clinically relevant difference in urine sodium concentration of 10 ± 2 mEq/L between baseline and the end of the surgery [18], a minimum number of 70 patients was required to ensure a study power of 0.80, given a confidence level of 0.05. We considered a 5% increase in the total number of enrolled subjects, to account for possible dropouts, protocol deviations or missing data. A data analysis and statistical plan was written and filled with the institutional review board before data were accessed. Continuous data are reported as mean ± SD or median [IQR], as appropriate; categorical data are reported as number (%). One-way Analysis of Variance (ANOVA) for repeated measures or Friedman test were performed to assess differences within the three measurement timepoints in terms of plasma and urine acid-base variables, gas exchange and respiratory mechanics. A *post-hoc* analysis with Bonferroni correction was performed for multiple comparisons. To investigate the differential effect of an increased intra-abdominal pressure, which may increase sodium retention, and of an increased PaCO_2_, which is thought to increase chloride excretion, we perfomed a two-ways ANOVA considering an high or low sodium excretion fraction (FeNa – based on the median value of FeNa at T0) as *between* effect, the measurement time points as *within* effect and patients as *random* effect. A p value of < 0.05 was considered as statistically significant. Statistical analyses and figures were performed using R Studio (RStudio. Integrated Development for R. RStudio, PBC, Boston, USA).

## Results

A total of 73 consecutive patients were enrolled and resulted available for the final analysis. Their baseline characteristics are presented in Table 1. Of these patients, 53 (39%) had a previous history of hypertension and 18 (13%) had a previous history of diabetes mellitus. Seventy (96%) underwent colorectal robotic-assisted surgery and 3 (4%) underwent gynecological robotic-assisted surgery.

**Table 1:** Baseline characteristics of the study population. ASA: American society of anesthesiologists

	*n* = 73
Age, years	68 [58–75]
Female sex, *% (n)*	50 (36)
Weight, *kg*	68 [61–80]
Body mass index, *kg/m*^*2*^	24 [22–28]
ASA Classification, *% (n)* 1 2 3	5 (4) 53 (39) 42 (30)
History of, *% (n)* Hypertension Diabetes mellitus Coronary artery disease Cerebrovascular disease	53 (39) 18 (13) 6 (4) 7 (5)
Type of surgical intervention, *% (n)* Colorectal Gynaecological	96 (70) 4 (3)
Plasma creatinine, *mg/dL*	0.6 [0.6–0.8]
Anesthesia time, *min*	310 [283–403]
Surgical time, *min*	255 [228–256]

### Plasma acid-base equilibrium

Both apparent and effective strong ion difference (SID) did not change throughout the study. However, a slight decrease in plasma [Na^+^] were observed at T1 and T2 compared to T0 and a slight increase in plasma [K^+^] at T1 and T2 compared to T0 (see Table 2, Figure S2). At the end of surgery, albumin levels were significantly lower, and phosphates levels were significantly higher than at T0 (see Table 2; Fig. 2). As a resultant, A_TOT_ increased a T1 as compared to T0, while returning to baseline values at T2. Strong ion gap did not change throughout the study,

**Table 2:** Time-course of ventilatory settings, respiratory mechanics, gas exchange and plasma acid-base variables after general anesthesia induction (T0), 2 h after the start of pneumoperitoneum (T1) and at the end of surgery, after pneumoperitoneum interruption (T2). PEEP: positive end-expiratory pressure; FiO_2_: administered oxygen fraction; PaCO_2_: arterial carbon dioxide partial pressure; PaO_2_: arterial oxygen partial pressure; [HCO_3_^−^]: arterial bicarbonate concentration; [Lac]: lactate; SID: strong ion difference; A_TOT_: total non-carbonic weak acid concentration; SIG: strong ion gap. *: *p* < 0.05 vs. T0; °: *p* < 0.05 vs. T1

	General anesthesia T0	During PnP T1	End of surgery T2	*p*
Tidal volume, mL	500 [450–520]	500 [450–520]	500 [450–520]	0.113
Respiratory rate, *bpm*	12 [11–12]	13 [12–14]*	14 [12–14]*	*< 0.001*
Minute ventilation, *L min*^*− 1*^	5.8 [5.2–6.4]	6.6 [5.8–7.1]*	6.6 [5.9–7.5]*	*< 0.001*
PEEP, *cmH*_*2*_*O*	5 [5–5]	5 [5–5]	5 [5–5]	*0.829*
FiO_2_	0.4 [0.4–0.4]	0.4 [0.4–0.4]	0.4 [0.4–0.4]	*0.112*
Peak pressure, *cmH*_*2*_*O*	17 [15–19]	24 [21–27]*	19 [16–22]*°	*< 0.001*
Plateau pressure, *cmH*_*2*_*O*	13 [12–15]	18 [16–20]*	14 [12–16]*°	*< 0.001*
Driving pressure, *cmH*_*2*_*O*	8 [7–10]	13 [11–15]*	9 [7–11]*	*< 0.001*
Airway resistance, *cmH*_*2*_*O L*^*− 1*^ *sec*^*− 1*^	14 [11–18]	17 [13–20]*	15 [12–18]	*< 0.001*
Respiratory system elastance, *cmH*_*2*_*O L*^*− 1*^	17 [13–20]	26 [22–31]*	18 [15–23]*°	*< 0.001*
Mechanical power, *J min*^*− 1*^	7.5 [6.5–8.9]	11.2 [9.4–13.5]*	9.5 [7.9–12.1]*°	*< 0.001*
Arterial pH	7.41 [7.37–7.44]	7.36 [7.33–7.38]*	7.37 [7.35–7.40]*°	*< 0.001*
PaCO_2,_ *mmHg*	41 [37–45]	45 [42–51]*	43 [41–46]*°	*< 0.001*
PaO_2,_ *mmHg*	163 [123–193]	156 [123–179]	152 [127–173]	*0.554*
EtCO_2_, *mmHg*	35 [33–37]	39 [35–40]*	35 [33–37]°	*< 0.001*
Ventilatory ratio	0.9 [0.7–1.0]	1.1 [0.9–1.3]*	1.1 [0.9–1.3]*	*< 0.001*
[HCO_3_^−^], *mMol L*^*− 1*^	25.2 [23.9–26.8]	25 [24–28]	25 [24–27]	*0.367*
Standard base excess, *mMol L*^*− 1*^	0.5 [-0.6–2.8]	1.7 [0.4–3.4]*	0.9 [-0.1–2.9]°	*0.047*
Plasma [Na^+^], *mEq L*^*− 1*^	137 [136–139]	137 [136–138]*	136 [135–138]*°	*< 0.001*
Plasma [K^+^], *mEq L*^*− 1*^	3.7 [3.5–4.0]	4.0 [3.7–4.3]*	4.2 [2.9–4.6]*°	*< 0.001*
Plasma [Ca^+^], *mEq L*^*− 1*^	1.20 [1.18–1.21]	1.2 [1.19–1.22]	1.20 [1.18–1.22]	*0.381*
Plasma [Mg^+^], *mEq L*^*− 1*^	2.1 [2.0–2.2]	2.5 [2.2–2.9]*	2.5 [2.3–2.7]*°	*< 0.001*
Plasma [Cl^−^], *mEq L*^*− 1*^	105 [104–107]	105 [104–107]	105 [103–106]	*0.749*
Plasma [Lac], *mMol L*^*− 1*^	0.99 [0.96–1.06]	1.0 [0.9–1.1]	1 [0.9–1.1]	*0.157*
Plasma apparent SID, *mEq L*^*− 1*^	40 [38–41]	40 [39–41]	39 [38–41]	*0.065*
Plasma albumin, *g dL*^*− 1*^	3.4 [3.1–3.6]	3.4 [3.2–3.6]	3.2 [3–3.5]*°	*< 0.001*
Plasma phosphates, *mg dL*^*− 1*^	3.2 [2.9–3.7]	3.7 [3.3–4.3]*	4.0 [3.4–4.4]*°	*< 0.001*
A_TOT_, *mMol L*^*− 1*^	11.3 [10.3–12.0]	11.7 [10.9–12.6]*	11.2 [10.5–11.9]°	*0.002*
Plasma effective SID, *mEq L*^*− 1*^	36 [35–38]	37 [35–39]	36 [34–37]	*0.129*
Plasma SIG, *mEq L*^*− 1*^	3 [2–3]	3 [2–4]	3 [3–4]	*0.160*

### Urine production and urinary acid-base equilibrium

The total urine production from T0 to T2 was 400 [250–665] mL. Urine [Na^+^] and [Cl^−^] did not change throughout the study, while urine [K^+^] progressively significantly increased from T0 to T1 and T2. Accordingly, urinary anion gap significantly increased from 35 [26–42] at T0 and 35 [24–43] at T1 to 39 [28–53] mEq L^− 1^ at T2 (see Table 3). The application of pneumoperitoneum significantly reduced sodium absolute excretion during pneumoperitoneum application and at the end of the surgery compared to T0, and fractional sodium excretion at T2 as compared to T0. Norepinephrine requirement and infusion rates are reported in Table S1.

**Table 3:** Time-course of urine acid-base variables after general anesthesia induction (T0), 2 h after the start of pneumoperitoneum (T1) and at the end of surgery, after pneumoperitoneum interruption (T2). *: *p* < 0.05 vs. T0; °: *p* < 0.05 vs. T1

	General anesthesia T0	During PnP T1	End of surgery T2	*p*
Total infusion, mL	1000 [600–1100]	1600 [1200–2000]	2500 [1212–3425]	-
Differential urine output, *mL*	200 [100–360]	70 [30–200]*	100 [50–200]*	*0.021*
Urine [Na^+^], *mEq L*^*− 1*^	121 [91–148]	116 [88–144]	119 [86–150]	*0.293*
Absolute Na^+^ excretion, *mEq*	17 [8–29]	8 [3–21]*	10 [5–17]*	*0.041*
Na fractional excretion, *%*	1.20 [0.70–2.30]	1.30 [0.60–2.1]*	0.9 [0.6–1.8]*	*0.011*
Urine [K^+^], *mEq L*^*− 1*^	21 [14–34]	28 [19–40]*	34 [22–51]*°	*< 0.01*
Absolute K^+^ excretion, *mEq*	3 [2–5]	2 [1–5]	4 [1–7]	*0.062*
K fractional excretion, *%*	9.4 [5.7–12.7]	9.8 [7.3–13.8]	10 [6–15.7]	*0.837*
Urine [Cl^−^], *mEq L*^*− 1*^	101 [79–139]	108 [81–135]	109 [82–135]	*0.743*
Absolute Cl^−^ excretion, *mEq*	15 [7–25]	8 [2–17]	10 [5–18]	*0.186*
Cl fractional excretion, *%*	1.4 [0.9–2.4]	1.5 [0.8–2.2]	1.1 [0.7–2.0]	*0.169*
Urinary anion gap, *mEq L*^*− 1*^	35 [26–42]	35 [24–43]	39 [28–53]*°	*0.002*

### Patients with higher vs. lower sodium fractional excretion

The population was divided according to the median sodium fractional excretion value at T0 (1.20%). The two groups presented similar arterial pH, carbon dioxide and apparent SID values at T0 (Table S2, Figure S3). Patients with lower sodium fractional excretion had lower urinary chloride excretion than patients with higher sodium fractional excretion (Table S3). During pneumoperitoneum application and at the end of surgery, arterial pH was significantly lower and PaCO_2_ was significantly higher in both groups compared to T0, without any difference between groups. The behaviour of sodium and chloride fractional excretion were similar within the three timepoints between patients with higher or lower sodium fractional excretion at T0 (Table S3).

### Ventilatory settings and respiratory mechanics

**Fig. 1:**
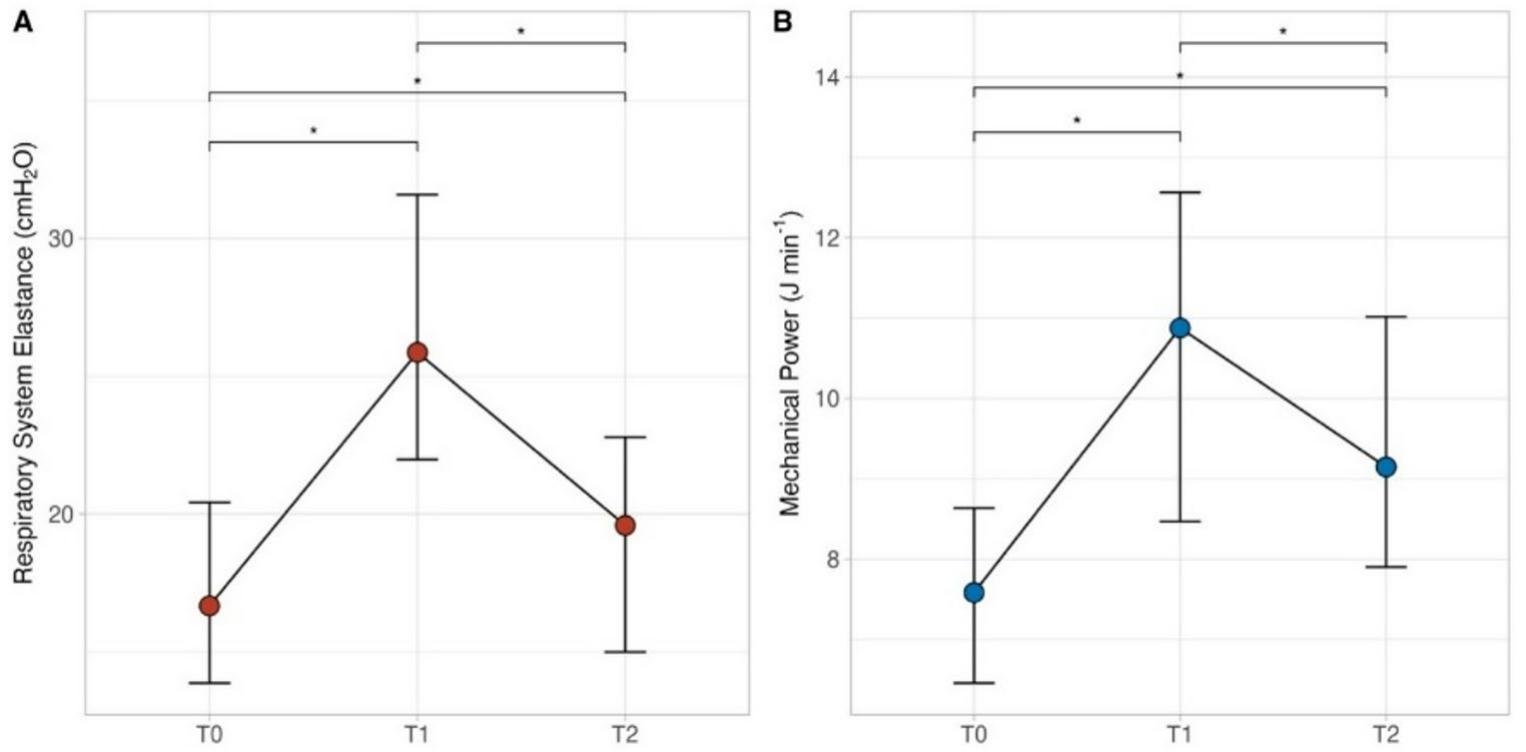
Timecourse of repiratory system elastance (**A**) and mechanical power (**B**) within measurements timepoints, after general anesthesia induction (T0), 2 hours after the start of pneumoperitoneum (T1) and at the end of the surgery after pneumoperitoneum interruption (T2). *: *p* < 0.050

After anesthesia induction (T0), tidal volume, PEEP and inspiratory oxygen fraction were 500 [450–520] mL, 5 [5] cmH_2_O and 0.40 [0.40–0.40], respectively. These values remained unchanged throughout the study (Table 2). The respiratory rate was significantly higher during pneumoperitoneum administration (T1) and at the end of the surgery (T2) as than at T0 (12 [13, 14] and 14 [12–14] vs. 12 [11, 12], *p* < 0.001). The administration of pneumoperitoneum resulted in significantly higher airway peak and plateau pressure at T1 compared to T0 (24 [21–27] vs. 17 [15–19] cmH_2_O and 18 [16–20] vs. 13 [12–15] cmH_2_O, respectively), which resulted in an increase in total airway resistance and in respiratory system elastance. At the end of surgery, both peak and plateau airway pressure decreased, although they remained significantly higher than at T0. Mechanical power increased significantly after 2 h from pneumoperitoneum administration (T1), then decreased at the end of surgery though remaining higher than at T0 (see Table 2; Fig. 1). On the other hand, sodium absolute excretion decreased from T0 until the end of surgery, regardless of pneumoperitoneum removal.

**Fig. 2:**
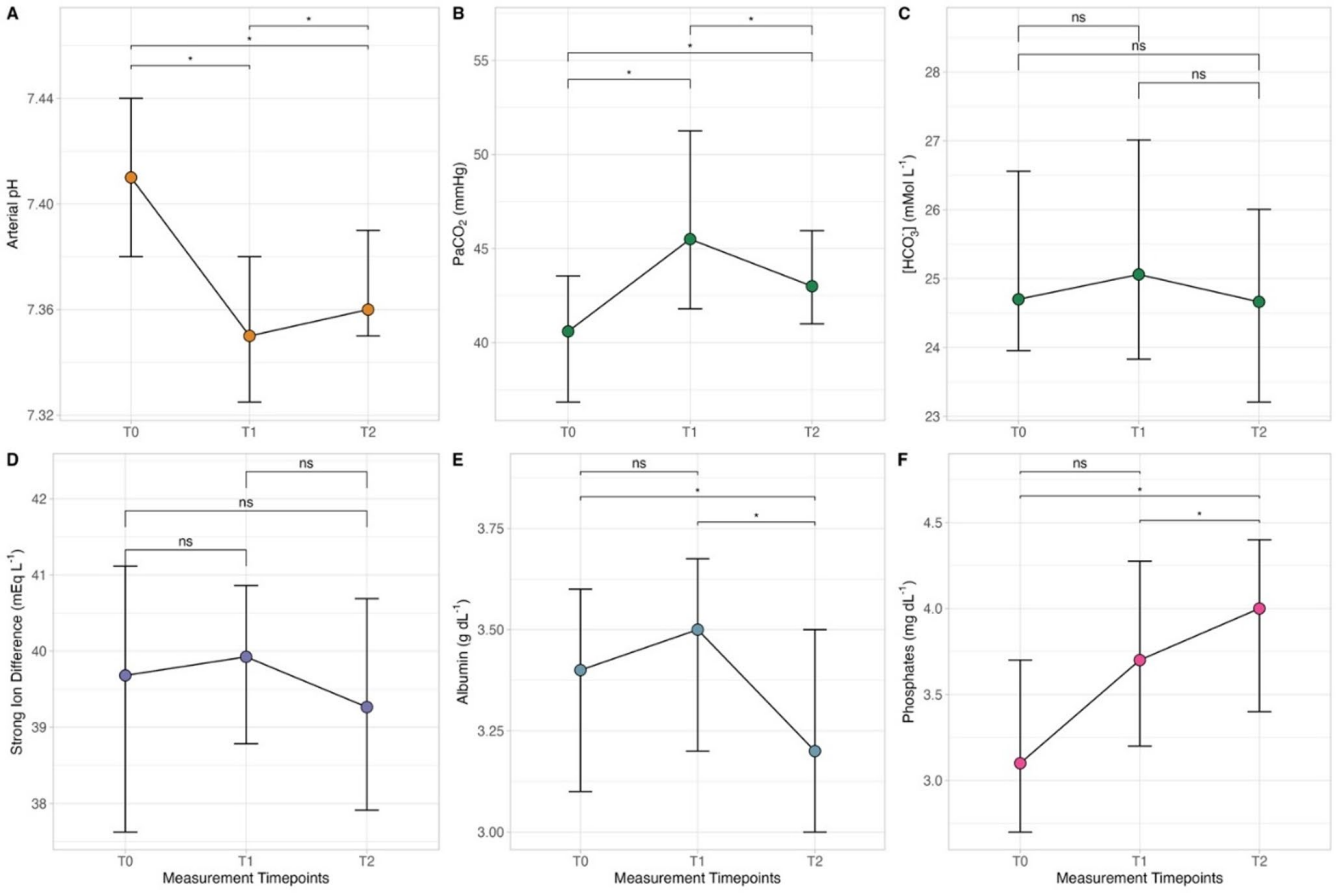
Timecourse of arterial pH (**A**), arterial carbon dioxide partial pressure (PaCO_2_, **B**), bicarbonate concentration (**C**), strong ion difference (**D**), plasma albumin concentration (**E**) and plasma phosphate concentration (**F**) within measurements timepoints, after general anesthesia induction (T0), 2 hours after the start of pneumoperitoneum (T1) and at the end of the surgery after pneumoperitoneum interruption (T2). *: *p* < 0.050

### Gas exchange

Arterial oxygenation did not change throughout the study. Pneumoperitoneum administration significantly increased the partial pressure of arterial carbon dioxide as compared to T0 (45 [42–51] vs. 41 [37–45] mmHg), resulting in a decrease in arterial pH. At the same time, it caused an increase in the ventilatory ratio at T1 compared to T0 (1.1 [0.9–1.3] vs. 0.9 [0.7–1.0]). At the end of surgery, both PaCO_2_ and the ventilatory ratio decreased significantly compared to T1 but remained higher than at T0, without any differences in terms of urinary chloride fractional excretion.

## Discussion

The major findings of this study, which evaluated the intraoperative effects of pneumoperitoneum during robotic-assisted colorectal and gynecological surgery, are: (1) arterial pH decreased during pneumoperitoneum application and at the end of the surgery, due to an increase in arterial carbon dioxide without any compensatory increase in urinary chloride excretion, (2) sodium absolute excretion reduced from general anesthesia induction to the end of the surgery after pneumoperitoneum removal, while airway peak and plateau pressure, respiratory system elastance and mechanical power significantly increased during pneumoperitoneum application and at the end of surgery, (3) patients with a lower sodium fractional excretion at general anesthesia induction showed a lower sodium and chloride fractional excretion during and after pneumoperitoneum application compared to patients with a higher sodium fractional excretion at general anesthesia induction; however, the behaviour of sodium and chloride fractional excretion were similar within the three timepoints between the two groups.

### Acid-base equilibrium

Pneumoperitoneum often results in hypercapnia and respiratory acidosis due to CO₂ absorption through the peritoneum [19]. Early compensation involves non-carbonic buffering by plasma proteins and phosphates, whereas renal mechanisms intervene later. This renal response consists of ammonium and chloride excretion. According to the Henderson–Hasselbalch approach, chloride excretion promotes bicarbonate reabsorption and increases plasma bicarbonate, while according to Stewart’s approach it increases plasma SID [20]. Renal compensation begins 30–60 min after the hypercapnic stimulus and involves dissociation between sodium reabsorption and chloride excretion [21], leading to an increased SID through reduced plasma chloride.

Beyond hypercapnia, pneumoperitoneum impairs haemodynamics by compressing venous and arterial compartments, reducing venous return and increasing afterload. This may decrease cardiac output and renal blood flow, stimulating both sodium and chloride reabsorption without changing plasma SID. Reduced cardiac output and renal and splanchnic blood flow have also been associated with metabolic acidosis [7, 22–24]. Taura et al. showed that pneumoperitoneum at 15 mmHg induced lactic acidosis due to regional hypoxia [25], whereas Kwak et al. reported an intraoperative pH decrease due solely to respiratory factors, with no change in SID or lactate [19].

In this study, Stewart’s approach was applied, and both plasma and urinary components were assessed. A significant decrease in arterial pH during and after pneumoperitoneum was observed due to increased PaCO₂. Zadek et al. hypothesised that acute hypercapnia leads to an increase in plasma sodium concentration due to the entrance of free water into red blood cells, caused by an increased intracellular osmolarity [26]. Conversely, we did not expected variations in sodium concentration; however, we observed a slight reduction in plasma sodium concentration, probably because of the administration of hypotonic balanced solutions during surgery, dampening the sodium retention mechanism in response to increased intra-abdominal pressure. Moreover, we did not find the expected reduction in chloride concentration, nor an increase in plasma SID, likely due to the dominant haemodynamic effect of increased intra-abdominal pressure, leading to sodium and chloride reabsorption through mechanical and neuroendocrine mechanisms [27, 28]. Aldosterone and other hormones were not measured, although their renal effects are described mainly in cardiac surgery [29, 30].

To restore acid–base equilibrium, the primary cause of acidemia must be identified. In respiratory acidosis, CO₂ insufflation and pneumoperitoneum pressure should be reduced; in metabolic acidosis, cardiac output should be increased to restore abdominal perfusion [31].

Previous studies reported significant haemodilution during surgery, with decreased albumin after laparoscopy [18]. In our study, albumin decreased significantly, likely due to fluid administration. All patients received Ringer’s Lactate (mean 2500 mL; fluid balance + 1200 mL), containing sodium 130 mEq/L and chloride 109 mEq/L, with an in-vivo SID of 28 mEq/L. Dilution by Ringer’s Lactate reduces plasma apparent SID, because of its lower SID, and decreases albumin by hemodilution, producing opposing effects (metabolic acidosis and alkalosis) that likely counterbalanced each other [32]. Additionally, no accumulation of unmeasured anions occurred during surgery, as determined by the unchanged strong ion gap.

Urinary analysis showed a significant reduction in absolute and fractional sodium excretion during and after pneumoperitoneum, likely due to haemodynamic changes. A similar, non-significant trend was seen for chloride excretion.

Although increased PaCO₂ and reduced pH should increase chloride excretion, unchanged chloride output suggests a predominant effect of perceived volume depletion, inducing sodium and chloride retention during pneumoperitoneum. This is supported by similar retentive behaviour in patients with higher or lower baseline sodium fractional excretion.

Variations in the urinary anion gap provide indirect insight into renal ammonium excretion: a reduction suggests enhanced ammonium and chloride elimination, whereas an increase is generally associated with impaired acid excretion or effective volume depletion [33]. In our cohort, the urinary anion gap increased, suggesting reduced ammonium excretion due to an overriding effect of perceived volume depletion, with sodium and chloride retention and slight potassium increase. This may reflect underlying hyperaldosteronism induced by relative hypovolaemia.

### Respiratory mechanics and mechanical power

Regarding respiratory function, pneumoperitoneum causes a cranial displacement of the diaphragm, development of atelectasis—mainly in dependent lung regions—and an increase in lung and chest wall elastance [34, 35].

Papescou et al., comparing conventional laparoscopy and robotic-assisted surgery matched for anesthesia duration and pneumoperitoneum levels, found a significant increase in airway pressure and a decrease in compliance after pneumoperitoneum induction compared with post-induction of anesthesia [4]. n our study, airway peak and plateau pressures increased after pneumoperitoneum insufflation, with higher airway resistance and respiratory system elastance. These changes persisted until the end of surgery. Similar findings were reported in 91 patients undergoing laparoscopic abdominal surgery, with significant increases in airway resistance and respiratory system elastance [36]. The increase in driving pressure during pneumoperitoneum reflects both lung impairment (collapse and reduced lung volume) and increased chest wall elastance due to elevated intra-abdominal pressure [37].

To better characterize the interaction between ventilatory settings, respiratory mechanics and pneumoperitoneum, mechanical power has been proposed, using a framework similar to ARDS [38–40]. Mechanical power reflects the combined effects of tidal volume, driving pressure, PEEP, inspiratory flow and respiratory rate, as well as patient-specific respiratory mechanics [5, 41].

A retrospective cohort study of 262,723 patients undergoing general anesthesia (2008–2018) reported a mean mechanical power of 7.1 ± 3.5 J/min [39]. Intraoperative mechanical power was significantly higher in patients with postoperative respiratory failure than in those without (7.6 [5.6–10.1] vs. 6.6 [4.6–9.1] J/min). Higher mechanical power was associated with increased risk of reintubation, postoperative pulmonary complications and acute respiratory failure [38, 39].

In our study, pneumoperitoneum significantly increased mechanical power to 11.2 [9.4–13.5] J/min due to concurrent rises in dynamic (tidal volume, driving pressure) and resistive (airway resistance) components. Notably, this increase coincided with reduced absolute sodium excretion, which persisted even after pneumoperitoneum discontinuation and the subsequent decrease in mechanical power at the end of surgery, possibly indicating a direct mechanical effect on venous return and renal backpressure, inducing a prolonged stimulus for sodium retention.

### Gas exchange

The application of pneumoperitoneum to the abdomen affects gas exchange efficiency by promoting alveolar collapse. This results in lower arterial oxygenation and an increase in arterial carbon dioxide. The degree of this alteration is proportional to the level of the intra-abdominal pressure applied. An increase in arterial carbon dioxide can also arise from possible absorption by the peritoneal serosa, depending on the duration of surgery, the level of intra-abdominal pressure and the flux of carbon dioxide used [11, 12, 42, 43].

In the present study, the induction of pneumoperitoneum significantly increased arterial carbon dioxide, despite an increase in minute ventilation. Although pressure-controlled ventilation can deliver the same tidal volume with a lower peak inspiratory airway pressure, better dynamic compliance and higher CO_2_ clearance, it is not the preferred choice for ventilatory settings. The optimal ventilatory setting for abdominal robotic-assisted surgery is still a matter of debate [44, 45]. In this study, volume-controlled ventilation was used to avoid the risk of hypoventilation thus ensuring adequate minute ventilation and carbon dioxide clearance during general anesthesia.

### Limitations

This study has several limitations. First, we did not directly measure urinary ammonium concentration, but we had to rely on urinary anion gap to assume its variations throughout the study; a direct measurement could have allowed to precisely investigate both sodium and chloride ammonium excretion. Second, the measurement of circulating hormones could have allowed the precise quantification of neuroendocrine changes. Third, the observational design of our study did not allow us to draw definitive conclusions about the mechanistic relationship between pneumoperitoneum-induced hypercapnia and renal acid-base compensatory mechanisms.

## Conclusions

Robotic-assisted surgery is now the standard surgical approach surgical for many abdominal procedures. The induction of pneumoperitoneum causes an increase in arterial carbon dioxide with concomitant respiratory acidemia, without any compensatory increase in urinary chloride excretion and consequent plasma SID modification. However, pneumoperitoneum application reduces sodium excretion, as a consequence of a prevalent sodium retention compensatory mechanism to the perceived hypovolemia from the kidney, while increasing mechanical power. This sodium retention compensatory mechanism during and after pneumoperitoneum reduces both sodium and chloride absolute excretion, independently of baseline sodium fractional excretion.

## Supplementary Information

Below is the link to the electronic supplementary material.

Supplementary Material 1

## Data Availability

The dataset used in this study is available upon reasonable request from the corresponding author.

## Abbreviations

CO_2_: Carbon dioxide
PEEP: Positive-end expiratory pressure
ARDS: Acute respiratory distress syndrome
ASA: American society of anesthesiologists
FiO_2_: Inspired oxygen fraction
RR: Respiratory rate
EtCO_2_: End-tidal CO_2_ concentration
V_E_: Minute ventilation
PaCO_2_: Arterial carbon dioxide partial pressure
PaO_2_: Arterial oxygen partial pressure
HCO_3_^−^: Bicarbonate concentration
BE: Base excess
p[Na^+^]: Plasma sodium concentration
p[K^+^]: Plasma potassium concentration
p[Ca^++^]: Plasma calcium concentration
p[Cl^−^]: Plasma chloride concentration
p[Lac^−^]: Plasma lactate concentration
p[Mg^++^]: Plasma magnesium concentration
p[Na^+^]: Plasma sodium concentration
u[Na^+^]: Urine sodium concentration
u[K^+^]: Urine potassium concentration
u[Cl^−^]: Urine chloride concentration
MP: Mechanical power
VR: Ventilatory ratio
FeNa: Sodium fractional concentration
