# Supplementary material for: Acid–base balance and respiratory mechanics during robotic-assisted surgery: an observational study
Source: J Robot Surg. 2026 Apr 21;20(1):440. doi: 10.1007/s11701-026-03392-8 (PMC13095963; doi:10.1007/s11701-026-03392-8)
Supplement: Supplementary file 1 — Supplementary Material 1 [file 11701_2026_3392_MOESM1_ESM.docx]

**Respiratory mechanics, Mechanical Power and Acid–Base Balance During Robotic-assisted Surgery: An Observational Study**

Supplementary Material

Matteo Pitimada MD, Davide Chiumello MD, Alessandro Monte MD, Isabella Fratti MD, Federica Festa MD, Tommaso Pozzi MD and Silvia Coppola MD

# Supplementary Figures and Tables

## Supplementary Figures

**Figure S1**. Flow chart of the study protocol. PnP : pneumoperitoneum.


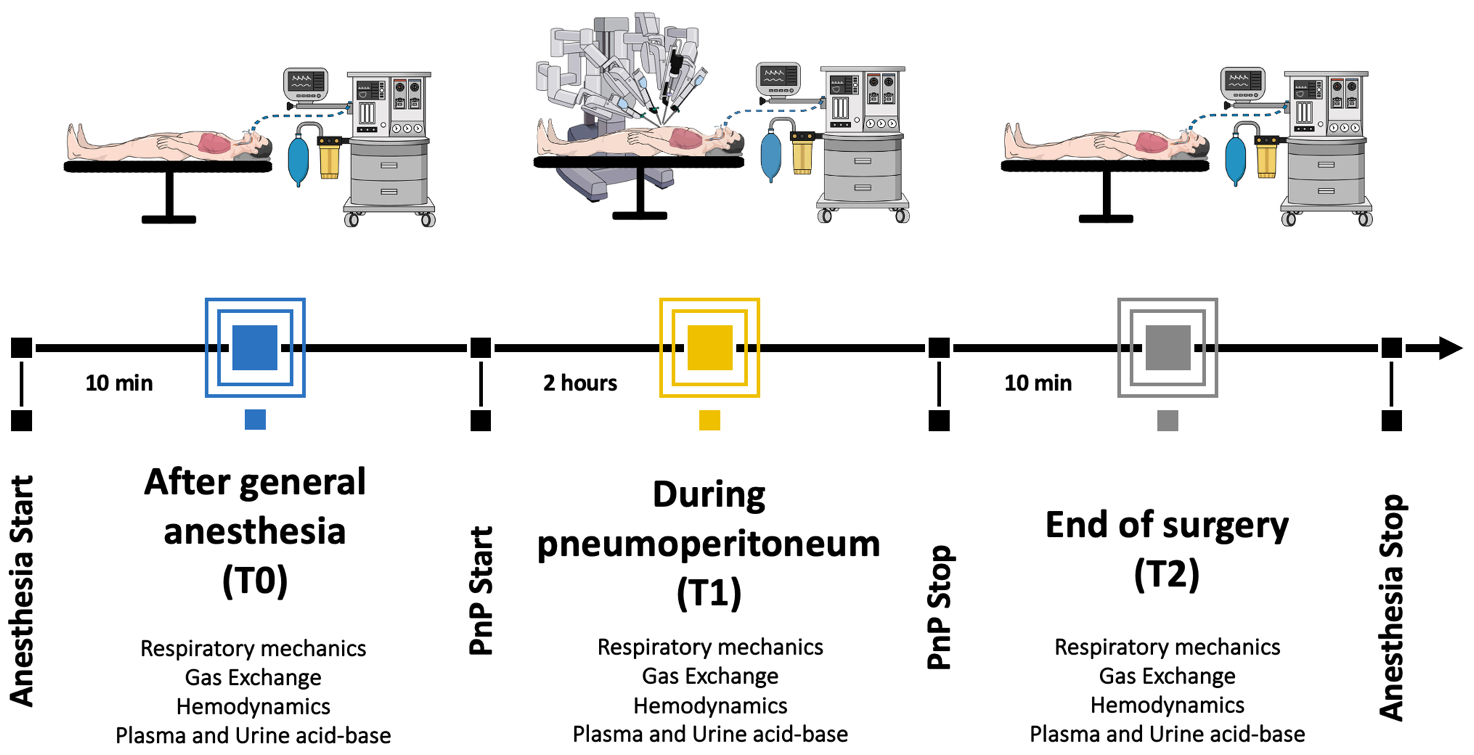


**Figure S2.** Timecourse of strong ion difference components, *i.e.,* plasma sodium (A), potassium (B), calcium (C), magnesium (D), chloride (E) and lactate (F) concentration, within measurements timepoints, after general anesthesia induction (T0), 2 hours after the start of pneumoperitoneum (T1) and at the end of the surgery after pneumoperitoneum interruption (T2). *: *p* < 0.050.

**
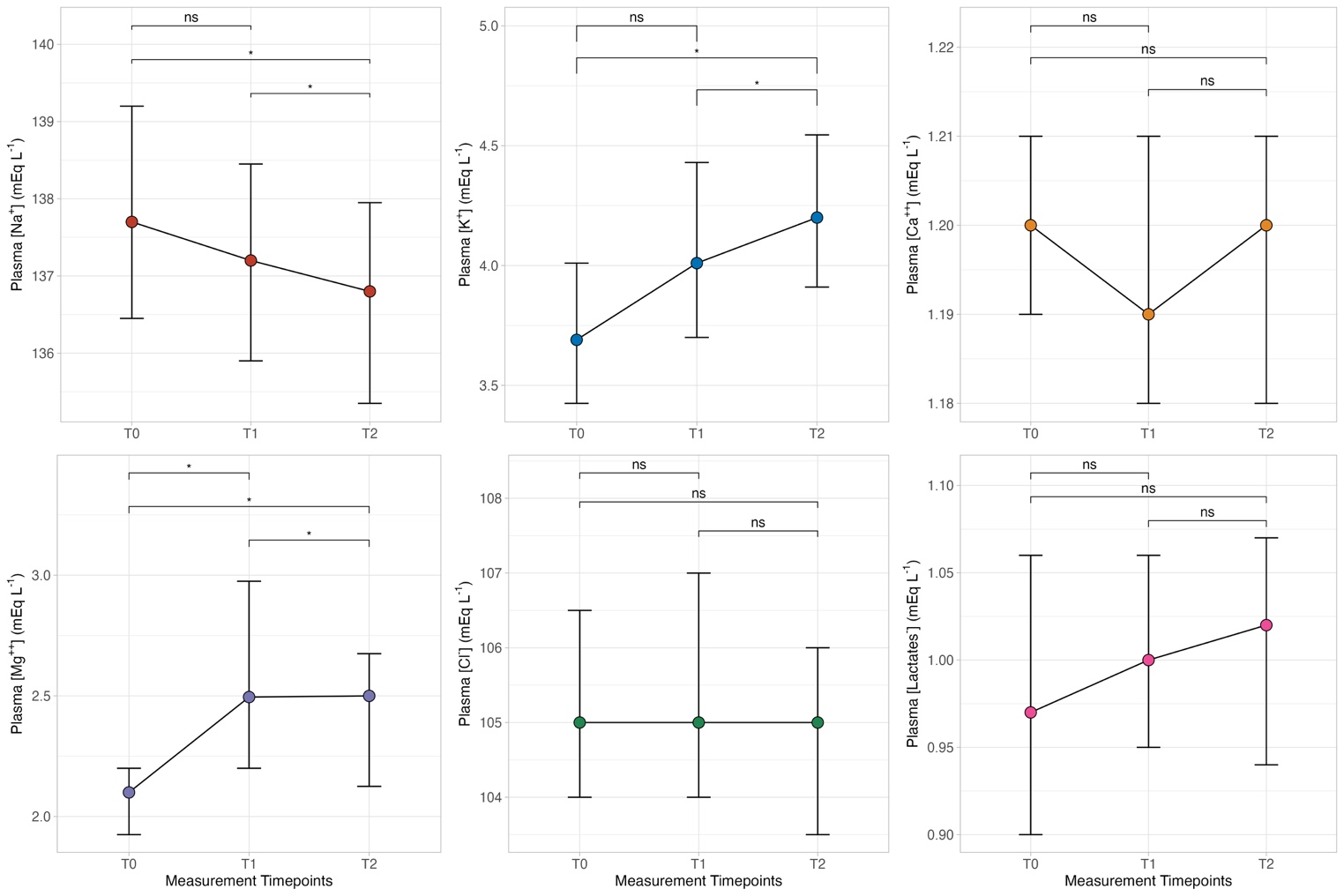
**

**Figure S3.** Timecourse of arterial pH (A), arterial carbon dioxide partial pressure (PaCO_2_ - B) and strong ion difference (C) after general anesthesia induction (T0), 2 hours after the start of pneumoperitoneum (T1) and at the end of surgery, after pneumoperitoneum interruption (T2) according to sodium fractional excretion (FeNa - higher as red dots *vs* lower than 1.20% as blue dots).

**
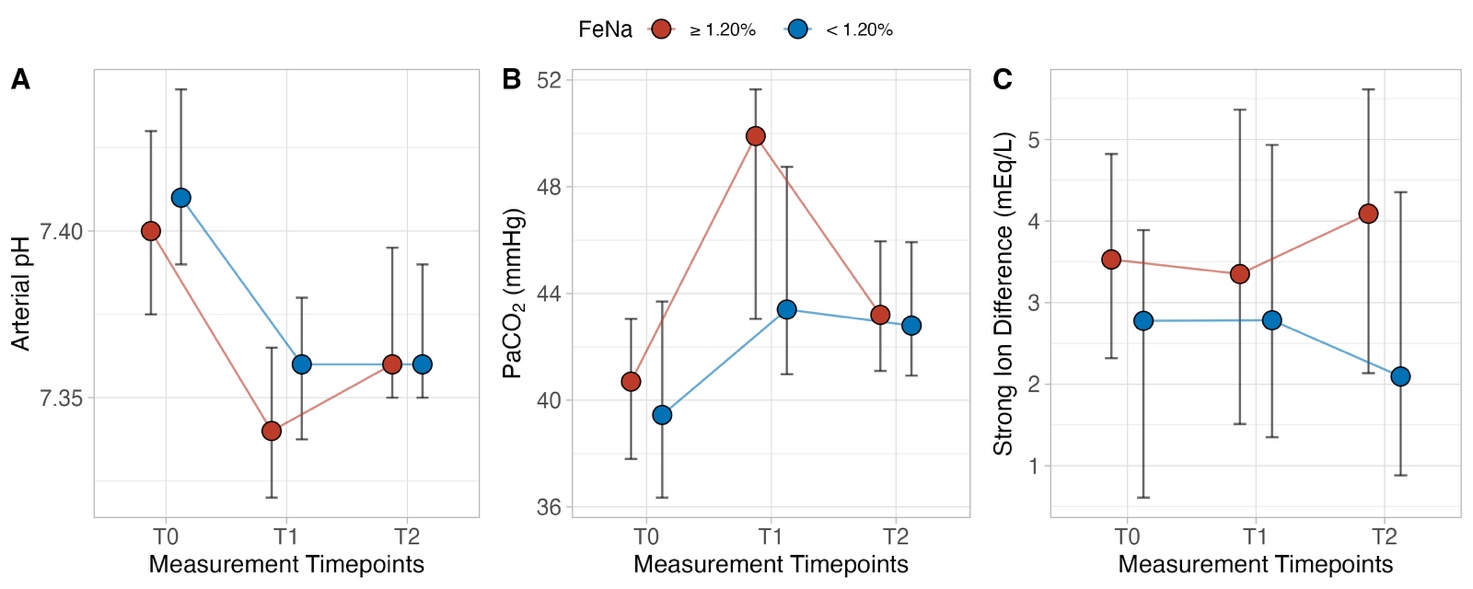
**

## Supplementary Tables

Table S1. Norepinephrine requirement and dose after general anesthesia induction (T0), 2 hours after the start of pneumoperitoneum (T1) and at the end of surgery, after pneumoperitoneum interruption (T2). *: *p* < 0.05 *vs* T0; °: *p* < 0.05 *vs* T1.

|  | General anesthesia T0 | During  PnP  T1 | End of surgery  T2 | *P* |
| --- | --- | --- | --- | --- |
| Norepinephrine requirement, *% (n)* | 19 (12) | 22 (14) | 19 (12) | *0.641* |
| Norepinephrine infusion rate, 𝜇*g/kg/min* | 0.05 [0.05 – 0.05] | 0.04 [0.03 – 0.07] | 0.07 [0.04 – 0.10] | *0.378* |

*Norepinephrine requirement differences were assessed by Cochran’s Q test.*

**Table S2**. Time-course of ventilatory settings, respiratory mechanics, gas exchange and plasma acid base variables after general anesthesia induction (T0), 2 hours after the start of pneumoperitoneum (T1) and at the end of surgery, after pneumoperitoneum interruption (T2) according to sodium fractional excretion (FeNa - higher *vs* lower than 1.20%). PEEP: positive end-expiratory pressure; FiO_2_: administered oxygen fraction; PaCO_2_: arterial carbon dioxide partial pressure; PaO_2_: arterial oxygen partial pressure; [HCO_3_^-^]: arterial bicarbonate concentration; [Lac]: lactate; SID: strong ion difference. *: *p* < 0.05 *vs* T0; °: *p* < 0.05 *vs* T1.

|  | FeNa  < 1.20% | FeNa  ≥ 1.20% | *p_GROUP_* | *p_TIME_* | *p_INTER_* |
| --- | --- | --- | --- | --- | --- |
| Tidal Volume, mL  *General anesthesia – T0*  *During PnP – T1*  *End of surgery – T2* | 500 [480 – 530]  500 [460 – 530]  500 [480 – 560] | 500 [430 – 530]  500 [450 – 540]  500 [460 – 540] | *0.234* | *0.105* | *0.879* |
| Respiratory rate  *General anesthesia – T0*  *During PnP – T1*  *End of surgery – T2* | 12 [12 – 12]  14 [12 – 14]*  14 [13 – 14]* | 12 [11 – 12]  13 [12 – 14]*  13 [12 – 14]* | *0.315* | *<0.001* | *0.643* |
| Minute ventilation, *L min^-1^*  *General anesthesia – T0*  *During PnP – T1*  *End of surgery – T2* | 6.0 [5.4 – 6.7]  6.6 [6.1 – 7.3]*  7.0 [6.3 – 7.8] * | 5.8 [5.5 – 6.7]  6.6 [5.8 – 7.5]*  6.4 [5.9 – 7.8]* | *0.143* | *<0.001* | *0.901* |
| PEEP, *cmH_2_O*  *General anesthesia – T0*  *During PnP – T1*  *End of surgery – T2* | 5 [5 – 5]  5 [5 – 5]  5 [5 – 5] | 5 [5 – 5]  5 [5 – 5]  5 [5 – 5] | *0.362* | *0.560* | *0.100* |
| FiO_2_  *General anesthesia – T0*  *During PnP – T1*  *End of surgery – T2* | 0.4 [0.4 – 0.4]  0.4 [0.4 – 0.4]  0.4 [0.4 – 0.4] | 0.4 [0.4 – 0.4]  0.4 [0.4 – 0.4]  0.4 [0.4 – 0.4] | *0.948* | *0.507* | *0.663* |
| Peak pressure, *cmH_2_O*  *General anesthesia – T0*  *During PnP – T1*  *End of surgery – T2* | 17 [15 – 20]  24 [21 – 16]*  19 [17 – 22]*§ | 16 [15 – 19]  24 [21 – 26]*  19 [16 – 21]*§ | *0.839* | *<0.001* | *0.641* |
| Plateau pressure, *cmH_2_O*  *General anesthesia – T0*  *During PnP – T1*  *End of surgery – T2* | 14 [12 – 16]  18 [17 – 20]*  15 [12 – 16]§ | 12 [12 – 15]  17 [16 – 20]*  14 [12 – 15]§ | *0.519* | *<0.001* | *0.902* |
| Driving pressure, *cmH_2_O*  *General anesthesia – T0*  *During PnP – T1*  *End of surgery – T2* | 9 [7 – 11]  13 [12 – 15]*  10 [7 – 11]§ | 7 [7 – 9]  13 [11 – 15]*  9 [8 – 10]§ | *0.373* | *<0.001* | *0.997* |
| Airway resistance, *cmH_2_O L^-1^ sec^-1^*  *General anesthesia – T0*  *During PnP – T1*  *End of surgery – T2* | 13 [10 – 16]  17 [12 – 19]  14 [12 – 17] | 14 [12 – 18]  18 [14 – 20]*  15 [12 – 19] | *0.321* | *<0.001* | *0.667* |
| Respiratory system elastance, *cmH_2_O L^-1^*  *General anesthesia – T0*  *During PnP – T1*  *End of surgery – T2* | 17 [13 – 21]  25 [22 – 30]*  18 [15 – 22]§ | 16 [14 – 19]  26 [23 – 30]*  17 [15 – 23]§ | *0.931* | *<0.001* | *0.984* |
| Mechanical power, *J min^-1^*  *General anesthesia – T0*  *During PnP – T1*  *End of surgery – T2* | 7.6 [6.7 – 9.1]  11.5 [9.2 – 13.1]*  10.2 [8.0 – 12.4]*§ | 7.4 [6.1 – 8.4]  10.9 [9.4 – 13.1]*  8.9 [7.6 – 12.0]*§ | *0.534* | *<0.001* | *0.731* |
| Arterial pH  *General anesthesia – T0*  *During PnP – T1*  *End of surgery – T2* | 7.41 [7.38 – 7.44]  7.36[7.34 – 7.38]*  7.36 [7.35 – 7.40]* | 7.41 [7.36 – 7.43]  7.35 [7.32 – 7.38]*  7.38 [7.35 – 7.40]*§ | *0.889* | *<0.001* | *0.976* |
| PaCO_2_*, mmHg*  *General anesthesia – T0*  *During PnP – T1*  *End of surgery – T2* | 41 [38 – 45]  43 [42 – 48]*  43 [41 – 46] | 41 [38 – 45]  47 [44 – 52]*  44 [41 – 47] | *0.691* | *0.010* | *0.857* |
| PaO_2,_ *mmHg*  *General anesthesia – T0*  *During PnP – T1*  *End of surgery – T2* | 158 [116 – 183]  154 [119 – 177]  154 [122 – 178] | 163 [135 – 181]  155 [126 – 180]*  139 [127 – 167]* | *0.932* | *0.067* | *0.010* |
| EtCO_2_, *mmHg*  *General anesthesia – T0*  *During PnP – T1*  *End of surgery – T2* | 36 [34 – 37]  38 [35 – 40]  35 [33 – 37]§ | 33 [32 – 37]  39 [35 – 41]*  36 [33 – 38]§ | *0.777* | *<0.001* | *0.145* |
| Ventilatory ratio  *General anesthesia – T0*  *During PnP – T1*  *End of surgery – T2* | 0.9 [0.8 – 1.0]  1.0 [0.9 – 1.3]*  1.0 [0.9 – 1.3]* | 0.9 [0.8 – 1.0]  1.1 [1.0 – 1.4]*  1.1 [0.9 – 1.3]* | *0.639* | *<0.001* | *0.434* |
| [HCO_3_^-^], *mMol L^-1^*  *General anesthesia – T0*  *During PnP – T1*  *End of surgery – T2* | 25 [24 – 28]  25 [23 – 27]  25 [24 – 28] | 26 [24 – 27]  26 [25 -28]  25 [24 – 27] | *0.698* | *0.952* | *0.911* |
| Standard Base Excess, *mMol L^-1^*  *General anesthesia – T0*  *During PnP – T1*  *End of surgery – T2* | 0.6 [-0.8 – 3.7]  0.9 [-0.8 – 3.3]  0.8 [-0.1 – 3.2] | 1.0 [-0.5 – 2.8]  2.3 [1.3 – 3.8]  1.0 [0.0 – 2.8] | *0.712* | *0.901* | *0.899* |
| Plasma [Na^+^], *mEq L^-1^*  *General anesthesia – T0*  *During PnP – T1*  *End of surgery – T2* | 137 [136 – 138]  137 [135 – 138]  136 [134 – 137] | 138 [136 – 140]  137 [136 – 138]  137 [136 – 138] | *0.113* | *0.814* | *0.695* |
| Plasma [K^+^], *mEq L^-1^*  *General anesthesia – T0*  *During PnP – T1*  *End of surgery – T2* | 3.6 [3.5 – 3.9]  4.1 [3.7 – 4.4]  4.2 [3.9 – 4.6] | 3.8 [3.6 – 4.0]  4 [3.8 – 4.3]  4.2 [4.0 – 4.5] | *0.869* | *0.563* | *0.167* |
| Plasma [Ca^+^], *mEq L^-1^*  *General anesthesia – T0*  *During PnP – T1*  *End of surgery – T2* | 1.20 [1.19 – 1.22]  1.21 [1.19 – 1.22]  1.20 [1.19 – 1.21] | 1.19 [1.18 – 1.21]  1.19 [1.18 – 1.21]  1.21 [1.19 – 1.21] | *0.712* | *0.288* | *0.454* |
| Plasma [Mg^+^], *mEq L^-1^*  *General anesthesia – T0*  *During PnP – T1*  *End of surgery – T2* | 2.1 [1.8 – 2.2]  2.5 [2.1 – 2.9]*  2.4 [2.3 – 2.5]* | 2.2 [2.0 – 2.5]°  2.6 [2.3 – 3.0]*  2.6 [2.4 – 2.7] | *0.031* | *<0.001* | *0.565* |
| Plasma [Cl^-^], *mEq L^-1^*  *General anesthesia – T0*  *During PnP – T1*  *End of surgery – T2* | 105 [103 – 106]  105 [103 – 107]  105 [103 – 106] | 105 [104 – 107]  105 [104 – 106]  105 [104 – 106] | *0.678* | *0.251* | *0.966* |
| Plasma [Lac], *mMol L^-1^*  *General anesthesia – T0*  *During PnP – T1*  *End of surgery – T2* | 1. [0.9 – 1.1] 2. [0.9 – 1.0]   1.0 [1.0 – 1.1] | 1.0 [0.9 – 1.0]  1.0 [1.0 – 1.0]  1.0 [0.9 – 1.1] | *0.748* | *0.102* | *0.512* |
| Plasma SID, *mEq L^-1^*  *General anesthesia – T0*  *During PnP – T1*  *End of surgery – T2* | 39 [37 – 41]  40 [39 – 40]  39 [38 – 40] | 40 [39 – 41]  40 [39 – 41]  40 [38 – 41] | *0.346* | *0.223* | *0.149* |
| Plasma albumin, *g dL^-1^*  *General anesthesia – T0*  *During PnP – T1*  *End of surgery – T2* | 3.4 [3.1 – 3.6]  3.5 [3.2 – 3.7]  3.3 [3.1 – 3.5] | 3.3 [3.1 – 3.6]  3.3 [3.2 – 3.5]  3.1 [3.0 – 3.3]°§ | *0.223* | *0.021* | *0.156* |
| Plasma phosphates, *mg dL^-1^*  *General anesthesia – T0*  *During PnP – T1*  *End of surgery – T2* | 3.3 [2.9 – 3.7]  3.9 [3.4 – 4.4]*  4.0 [3.4 – 4.5]* | 3.2 [2.7 – 3.8]  3.6 [3.2 – 4.1]*  3.9 [3.3 – 4.3]* | *0.367* | *<0.001* | *0.570* |

**Table S3**. Time-course of urinary acid base variables after general anesthesia induction (T0), 2 hours after the start of pneumoperitoneum (T1) and at the end of surgery, after pneumoperitoneum interruption (T2) according to sodium fractional excretion (higher *vs* lower than 1.20%). *: *p* < 0.05 *vs* T0; °: *p* < 0.05 *vs* T1.

|  | FeNa  < 1.20% | FeNa  ≥ 1.20% | *p_GROUP_* | *p_TIME_* | *p_INTER_* |
| --- | --- | --- | --- | --- | --- |
| Total infusion, *mL*  *General anesthesia – T0*  *During PnP – T1*  *End of surgery – T2* | 1300 [1080 – 1580]  2080 [1600 – 2360]  2700 [1920 –3920] | 1600 [1100 – 2000]  2350 [1670 – 2700]  3200 [2180 – 3950] | *-* | *-* | *-* |
| Differential urine output, *mL*  *General anesthesia – T0*  *During PnP – T1*  *End of surgery – T2* | 100 [50 – 230]  50 [30 – 100]  100 [50 – 190] | 250 [100 – 500]  100 [50 – 270]  130 [50 – 200] | *0.021* | *<0.001* | *0.434* |
| Urine [Na^+^], *mEq L^-1^*  *General anesthesia – T0*  *During PnP – T1*  *End of surgery – T2* | 119 [86 – 147]  113 [88 – 141]  107 [82 – 130] | 130 [112 – 150]  116 [98 – 144]  126 [100 – 158] | *0.076* | *0.413* | *0.212* |
| Absolute Na^+^ excretion, *mEq*  *General anesthesia – T0*  *During PnP – T1*  *End of surgery – T2* | 13 [7 – 23]  22 [12 – 38]  29 [20 – 68] | 32 [15 – 52]  39 [18 – 69]  58 [33 – 96] | *<0.001* | *<0.001* | *0.080* |
| Na fractional excretion, *%*  *General anesthesia – T0*  *During PnP – T1*  *End of surgery – T2* | 0.7 [0.5 – 1.0]  0.6 [0.4 – 1.0]  0.6 [0.4 – 0.9] | 2.3 [1.6 – 3.6]  2.0 [1.3 – 2.8]  1.6 [0.9 – 2.6] | *<0.001* | *0.199* | *0.186* |
| Urine [K^+^], *mEq L^-1^*  *General anesthesia – T0*  *During PnP – T1*  *End of surgery – T2* | 31 [24 – 39]  34 [27 – 49]  46 [30 – 61] | 16 [12 – 27]  19 [15 – 30]  28 [18 – 35] | *<0.001* | *0.089* | *0.503* |
| Absolute K^+^ excretion, *mEq*  *General anesthesia – T0*  *During PnP – T1*  *End of surgery – T2* | 3.6 [1.8 – 6.0]  6.4 [4.4 – 10.6]  13.8 [9.2 – 27.5] | 3.7 [2.7 – 6.4]  8.5 [3.5 – 12.3]  15.4 [5.3 – 23.7] | *0.745* | *<0.001* | *0.792* |
| K fractional excretion, *%*  *General anesthesia – T0*  *During PnP – T1*  *End of surgery – T2* | 6.6 [5.4 – 10.2]  8.4 [6.0 – 10.4]  8.4 [5.6 – 11.4] | 12.4 [8.3 – 18.8]  11.5 [8.7 – 16.1]  11.4 [9.1 – 17.3] | *0.079* | *0.831* | *0.785* |
| Urine [Cl^-^], *mEq L^-1^*  *General anesthesia – T0*  *During PnP – T1*  *End of surgery – T2* | 100 [70 – 147]  108 [85 – 132]  107 [79 – 131] | 107 [86 – 137]  107 [82 – 135]  120 [87 – 141] | *0.454* | *0.742* | *0.824* |
| Absolute Cl^-^ excretion, *mEq*  *General anesthesia – T0*  *During PnP – T1*  *End of surgery – T2* | 12 [7 – 21]  18 [11 – 35]  31 [19 – 61] | 22 [14 – 47]  35 [16 – 62]  50 [29 – 100] | *0.001* | *<0.001* | *0.183* |
| Cl fractional excretion, *%*  *General anesthesia – T0*  *During PnP – T1*  *End of surgery – T2* | 0.9 [0.6 – 1.1]  0.9 [0.5 – 1.2]  0.8 [0.6 – 1.1] | 2.4 [1.7 – 4.3]  2.2 [1.6 – 3.1]  2.0 [1.1 – 2.9] | *<0.001* | *0.951* | *0.547* |
